# Exposure to potentially traumatic events and PTSD symptomatology in Norwegian 11–13-year-olds: results from the Bergen Child Study

**DOI:** 10.1186/s13034-023-00578-y

**Published:** 2023-03-04

**Authors:** Annika Skandsen, Liv Sand, Martin H. Teicher, Ove Heradstveit, Tormod Bøe

**Affiliations:** 1 Department of Psychosocial Science, Faculty of Psychology, University of Bergen, Bergen, Norway; 2 Stavanger University Hospital, Gerd Ragna Bloch Thorsens Gate 25, Stavanger, Norway; 3 Regional Centre for Child and Youth Mental Health and Child Welfare, NORCE Norwegian Research Centre, Bergen, Norway; 4 Alcohol & Drug Research, Stavanger University Hospital, Stavanger, Norway; 5 Department of Psychiatry, Harvard Medical School, Boston, MA USA; 6 Developmental Biopsychiatry Research Program, McLean Hospital, Belmont, MA USA

**Keywords:** Trauma, PTSD, children, adolescents, Potentially traumatic events, Adverse childhood experiences, PTSD symptomatology, PTSD clusters

## Abstract

**Background:**

Exposure to potentially traumatic experiences (PTEs) is common among children and adolescents, but relatively little is known about the epidemiology of trauma and trauma-related psychopathology in children and youth. The present cross- sectional epidemiological study aimed to explore factors that is associated with posttraumatic stress symptoms (PTSS) in children.

**Method:**

Data stem from the Bergen Child Study, a series of cross-sectional multi-phase surveys of children born between 1993 and 1995 in Bergen, Norway. The sample used is from the second wave of the Bergen Child Study (BCS) conducted in 2006, a two-phase study. The study entailed a detailed psychiatric evaluation using the Development and well-being assessment (DAWBA). The DAWBA was administered to parents or caregivers and covered diagnostic areas, child and family background, and child strengths. A total of 2043 parents participated.

**Results:**

Out of the total sample, parents reported that 4.8% children had experienced PTEs at some point in their lives. The findings revealed current PTSS in 30.9% of children exposed to PTE, which was 1.5% of the total sample. None of the parents reported PTSS in their children over the threshold for diagnosing posttraumatic stress disorder (PTSD). The most common PTSS cluster was arousal reactivity (90.0%), followed by negative cognitions and mood (80%). The least frequent symptom cluster was intrusions (63.3%) and avoidance (60%). Children with PTSS were reported to live in families with significantly more family stressors (*p* = 0.001, *d* = 0.8) and had utilized significantly more sources for help relative to those without PTSS (*p* = 0.001, *d* = 0.75).

**Conclusion:**

The present population study on children revealed a lower prevalence rate of PTEs and PTSD than previous studies. It provided findings in the field of trauma on parent- reported PTSS and PTSD symptom clusters not restricted to the clinical level of PTSD. Lastly, it highlighted how family-life stressors and support differed between those who had PTSS and those with no PTSS.

## Introduction

Exposure to potentially traumatic experiences (PTEs) is common among children and adolescents [35]. A PTE involves exposure to an event involving threat, actual or perceived, to the life or physical safety of the individual, their loved ones or those around them, i.e., physical and psychological violence, sexual abuse, accidents/catastrophic events. It can be experienced on a single occasion or repeatedly [42].

Approximately 70% of the world’s adult population have been exposed to a potential traumatic life event [5, 30] and 33% of children aged 0–17 in the US have experienced at least one parent-reported PTE during their lifetime [41]. In Norway, Amstadter et al. [4] found that 25% of the participants had a lifetime PTE exposure, which is lower than previous world population studies. Norwegian studies of children and adolescents have mostly focused on specific PTEs such as physical, psychological and sexual abuse (e.g. [40], hence there is a need for additional knowledge on the frequency of exposure also to other types of PTEs in the Norwegian child population.

Several studies have documented negative psychological and physiological effects of having been exposed to PTEs. Among the negative consequences that have been described are mental ill health, sexual risk taking, interpersonal and self-directed violence, problematic alcohol and drug use, poor self-rated health, cancer, heart disease and respiratory disease (for a review, see [26]. The Adverse Childhood Experiences (ACE) study found that PTEs during childhood were strongly related to multiple risk factors for several of the leading causes of death in adults [16, 17].

Post-traumatic stress disorder (PTSD) is one of the most severe potential outcomes following PTE exposure [52]. PTSD is defined as a mental health condition that is triggered by either experiencing, witnessing, or hearing about a terrifying event. Symptoms may include intrusive memories of the event, avoidance, negative changes in thinking and mood, and changes in physical and emotional reactions [3]. In a meta-analysis by Alisic et al. [1] parents reported that 5% of children and adolescents developed PTSD after exposure to PTEs, but many more experience debilitating post-traumatic stress symptoms (PTSS) [19], sometimes referred to as *subthreshold* or *partial* PTSD [38]. Nevertheless, relatively few studies have investigated how PTE exposure in childhood is related to development of PTSD/subthreshold PTSD [35].

Whether PTE exposure leads to negative consequences may depend on characteristics of the PTE, such as the number of experiences. The greater the number of PTEs that a person is exposed to in childhood have consistently been found to be associated with more severe health problems and lower quality of adult life [16, 21]. There are fewer studies on this association in childhood, however existing findings support the association also among children [41].

Other factors that influence whether negative consequences occur following a child´s PTE exposure are vulnerability- and protective factors in those who are exposed. Factors such as level of family-life stress, quality of parenting practices, parent well-being and whether victims receive help following exposure are important variables that influence whether PTE exposure leads to negative consequences (i.e. [14, 51].

Family-life stressors such as separation/divorce and adaptation problems in school is found to increase the risk of PTSS [55, 58]. Williamson [57] postulate that the presence of higher level of general distress in a family with PTE exposed children may result in a parent being less available to their child during the post-traumatic period and consequently provide less healing posttraumatic care. This is supported by findings that family distress, amongst others was a predictor of severity of PTSS after PTEs in adolescents [14]. Nevertheless, Daniunaite [14] emphasizes that this impact of family distress can depend on various aspects such as the severity of the family-life stressor and the developmental age of the child when it occurs.

Parenting practices that provide coping assistance, emotional processing assistance and models healthy coping are found to be protective of those exposed to PTE [58]. However, the effects of positive (e.g., support and warmth) and negative (e.g., overprotection and hostility) parenting behaviour are so far consistent, but nevertheless small [57]. Positive parenting following a child´s exposure to PTE accounted for 2% of the variance in child PTSS development, while negative parenting behaviours accounted for 5.3% of the variance [57].

Children have also been found to be at increased risk of PTSS if their parents struggle with their own PTSS, high neuroticism and psychiatric illnesses [55, 58]. It would also be of interest to examine how a parent´s well-being in general might be associated with PTSS in their child, not limited to specific personality traits and illnesses.

Research on posttraumatic help have emphasized the beneficial effects of family support, social support, help from mental health care services [34, 58]. Receiving help after PTE exposure may nevertheless entail a great variety of support and interventions. The International society for traumatic stress studies (ISTSS) consequently generated 125 guidelines for prevention and treatment of PTSD based on 208 meta-analyses studies [7]. The recommendations ranged from strong to insufficient evidence to recommend. ISTSS emphasizes a continuous need for more research and detailed knowledge about the type of posttraumatic help which is beneficial after exposure to PTEs [7].

In sum, there is a need for improved knowledge about the epidemiology of trauma and trauma-related psychopathology in children [35], and specifically more research is needed to examine the relationship between number of PTEs and PTSD symptomatology in childhood*.* In addition, in PTE exposed children it is of interest to identify and understand in greater detail any associations that might render the child susceptible to PTSS. Accordingly, using data from the population-based Bergen Child Study, the aim of this study was to investigate the frequency of exposure to different PTEs in a community sample of children. Further, we aimed to assess the frequency of PTSS, PTSD diagnoses and PTSD symptom clusters in those with PTE exposure. Lastly, the objective was to examine if the PTE exposed children with PTSS vs those with no PTSS differed in terms of number of PTEs, family-life stressors, parental practices, parental well- being and support.

## Method

### Participants and procedure

Data stem from the Bergen Child Study, a series of cross-sectional multi-phase surveys of children born between 1993 and 1995 recruited from all public, private, and special schools in the city of Bergen, Norway. As the protocol and population have been detailed elsewhere, only a brief description will be provided in the current paper [9],https://www.norceresearch.no/en/projects/the-bergen-child-study). The sample used is from the second wave of the Bergen Child Study (BCS) conducted in 2006, a two-phase study, in which phase one included a questionnaire completed by children, parents and teachers. A total of 5791 children aged 11–13 years (M age = 11.8, SD = 0.8, 52% girls), their parents and teachers participated.

**Fig. 1:**
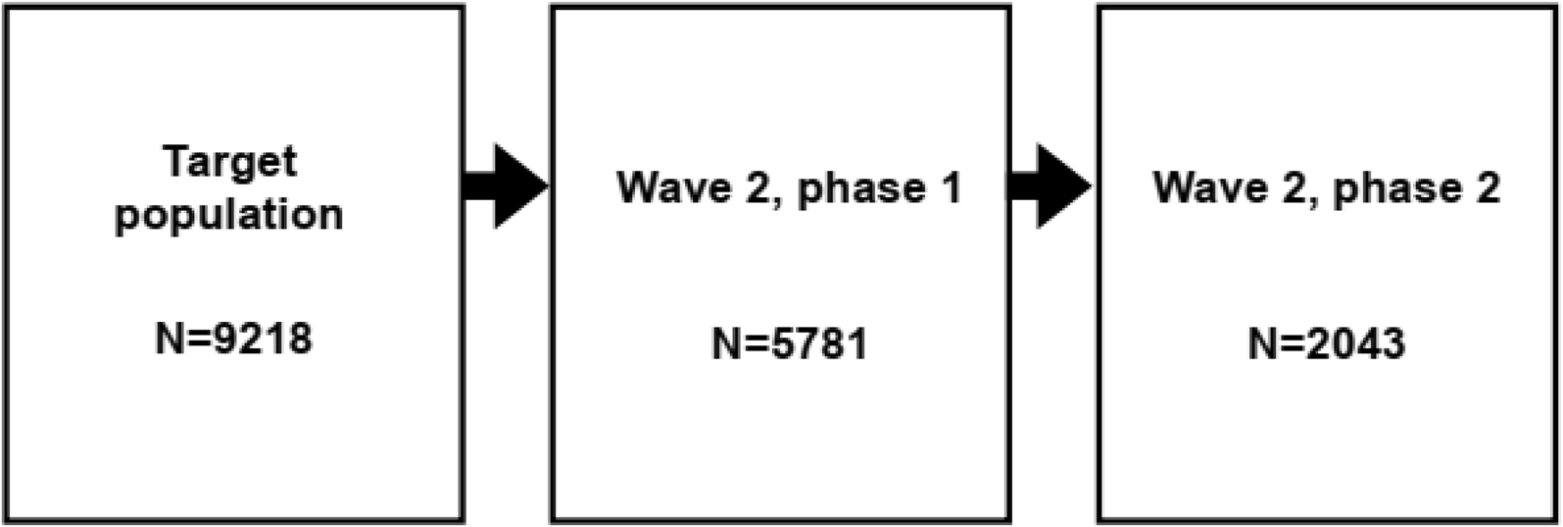
Overview of data sources used in the current project

**Fig. 2:**
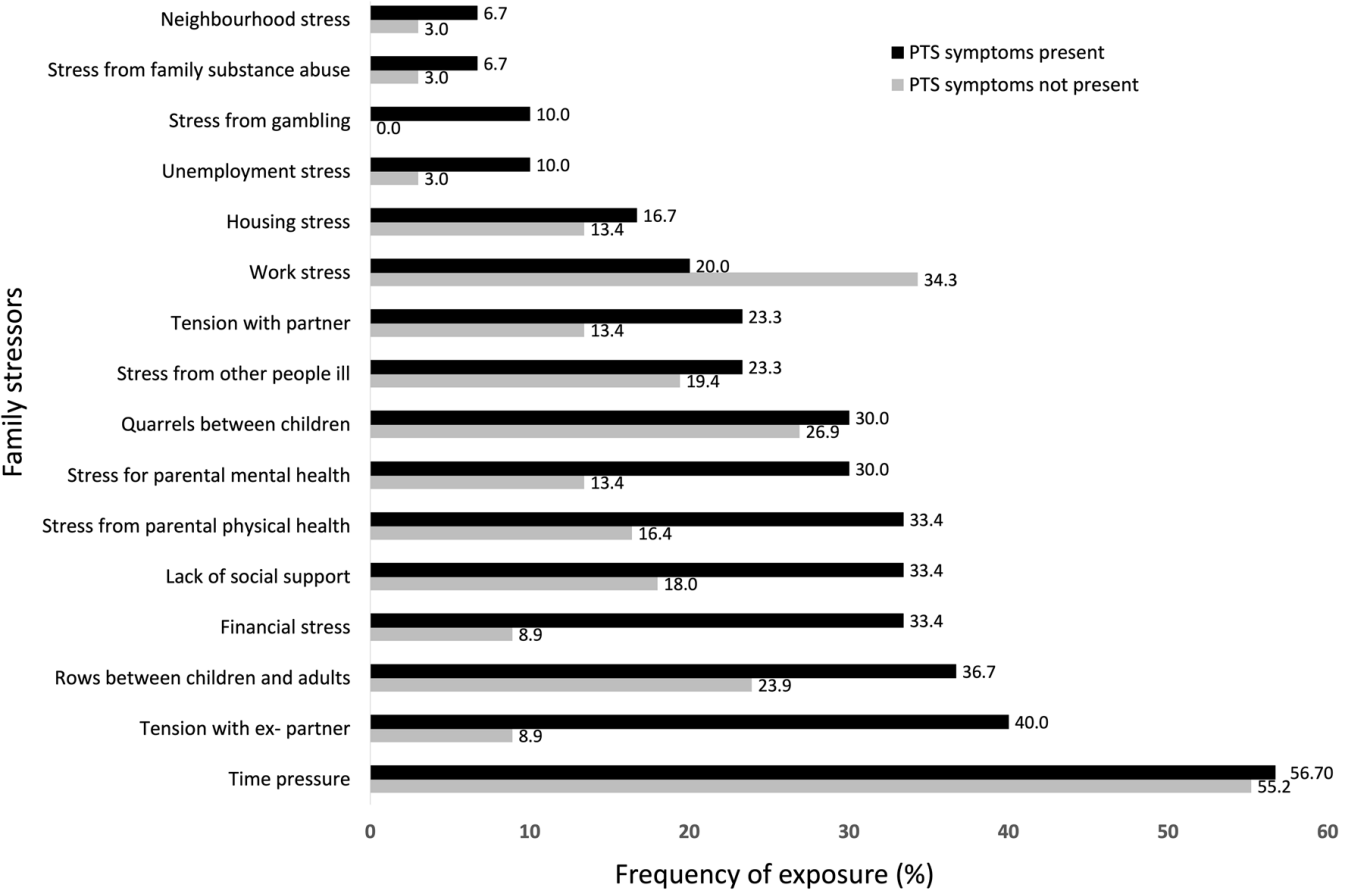
PTS symptoms and family stressors

All parents who participated in phase one were invited into phase two which was administered through a safe internet server which they logged on to. Phase two of the study entailed a detailed psychiatric evaluation using the Development and well-being assessment (DAWBA; [20], see Fig. 1. The DAWBA is a Web-based diagnostic interview that combines structured questions on symptoms and impairment with open-ended questions, the latter allowing the respondents to describe the child’s problems in their own words. The interview has a total of 16 sections. Depending on the number of problems reported, the time needed to complete the interview by caregivers may vary from 30 min to hours. If no problems are reported in the initial questions of a section, the interview becomes shorter due to skip-rules included in the web-based interview. The DAWBA administered to parents or caregivers covers diagnostic areas, child and family background, and child strengths. Our material included data obtained from parents’ report. A total of 2043 parents participated (c.f. Figure 1). All sections of the DAWBA interview were completed by 1364 individuals. A highly trained and experienced clinical rater (Fig. 2) assessed and assigned a psychiatric diagnose, with the help of the DAWBA program rater screens [23]. More details about the DAWBA is available on the website: http://dawba.info/a0.html.

The Regional Committee for Medical and Health Research Ethics in Western Norway approved the study.

### Representativeness of the phase two sample to the phase one sample

**Fig. 3:**
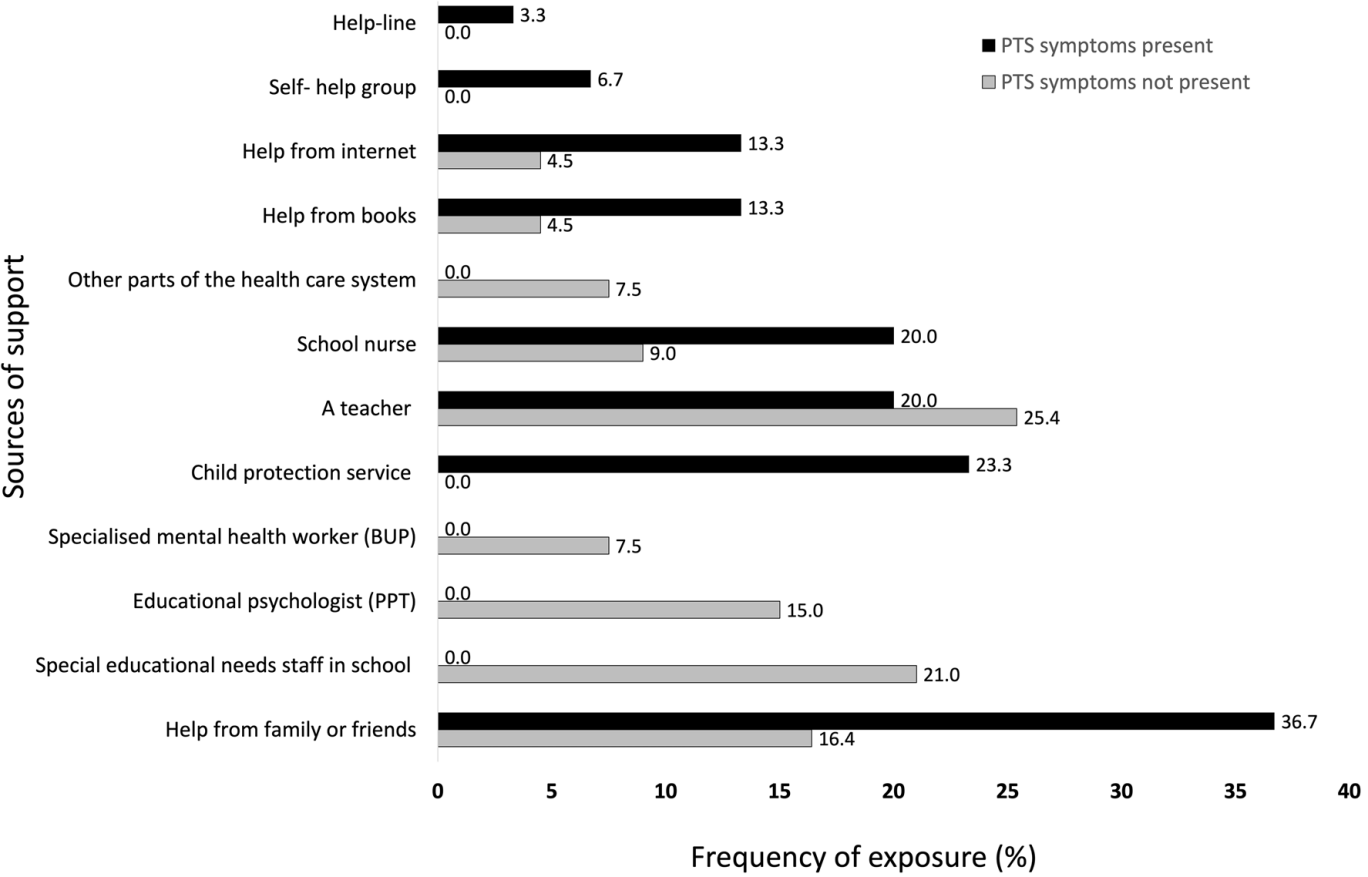
PTS symptoms and use of support

A previous study found that those who participated in the DAWBA psychiatric assessment sample (phase two) had better economic (Fig. 3) well-being, more highly educated parents and were slightly younger compared to those participating in the questionnaire phase only (phase one) [9]. In symptoms of mental health problems there were minor differences between participants in the two samples [9].

## Measures

### Demographic variables

Information on demographic variables was obtained by asking all participating parents and children about age and gender.

### Potential traumatic experiences (PTE) and post-traumatic stress symptoms (PTSS)

PTE and PTSS were measured with the sections of the DAWBA that assess post-traumatic stress disorder (PTSD) including the type and rate of PTEs a child had been exposed to. A list of eleven PTEs events were listed, to which the parent responded affirmative or not: child experienced serious accident; witnessed accident, sudden death; witnessed domestic abuse; child experienced sexual abuse; child experienced fire; child experienced attack or threat; child experienced rape; child witnessed attack; child experienced physical abuse; child experienced other disasters; other severe trauma. If PTEs were affirmed, parents were asked if the child experienced distress/behaviour change at the time of exposure *and* if the child experienced current distress (i.e., at the time of completing the questionnaire).

If any of the PTEs were affirmed and the child was experiencing current distress due to the experience, the parent was asked about current PTSD symptoms. The findings were examined on a symptom- and a diagnostic level. In line with the PTSD diagnostic criteria of the Diagnostic and statistical manual of mental disorders, 4th ed. [2], we categorized symptoms into four PTSD clusters; intrusions, avoidance, negative cognitions and mood, and arousal reactivity.

The DAWBA interview is known for having good psychometric qualities [8]. Interrater- reliability varied between Cohen’s kappa values of 0.69–0.82, depending on diagnostic category [8]. It has generated realistic prevalence estimates of mental disorders when used in public health services [24, 39] and has shown good ability to discriminate between children from community and clinical settings [20].

### Level of family distress

Level of family distress were assessed in the DAWBA. A list of sixteen family stressors were presented to the parents. Using options *no,* or *does not apply, a little* and *a lot*, parents indicated whether they currently were experiencing any of these events that made their family life stressful (e.g. “financial difficulties”, “home inadequate for family´s needs” and “unemployment”; [20]. A cumulative family stress measure was created by summing all items to which parents responded, *a lot*. A total score ranged from 0–17.

### Parenting practices

Parenting practices were examined with the Family Life Questionnaire (FaLQ [33] included as a section in the DAWBA. The FaLQ in DAWBA is a measure of current family functioning and consists of 14 items measuring four parenting facets: *Discipline*, which contains items related to punishment and inconsistent parenting practices, *Affirmation,* which measures aspects of the child- parent relationship (), *Rules,* the organization and structure within a family and *Special allowances* which relates to overinvolvement and under involvement from parents. The 14 items are divided into 5 subscales: Discipline (4 items, α = 0.5, ω = 0.4), Rules (2 items, α = 0.6), Support and encouragement (4 items, α = 0.6, ω = 0.6), Supervision (1 item), Special treatment (3 items, α = 0.4, ω = 0.4).

﻿Parents were asked to indicate how well the descriptions in the questionnaire apply to their child using four ordered response options (*not at all, a little, a medium amount and a great deal*). Last et al. [33] have previously found the FLQ to be a reliable measure of family function that seems sensitive to change (r = 0.73). They found that the test–retest reliability and internal consistency of the scales varied between moderate and very good, except for the *Discipline* items and *Special treatment* items which had poor internal consistency when grouped as a scale [33]. There was some evidence of validity, but this part of the study was limited by a lack of suitable comparators.

### Parents’ emotional well-being

Parents´ emotional well-being was examined by The everyday feeling questionnaire (EFQ); [54] This questionnaire uses 10 items (α = 0.90, ω = 0.90), to assess psychological distress and well-being of the parent (e.g. positive about the future; worried or tense; able to enjoy life; [54]. There were five response options *(none of the time, a little of the time, some of the time, most of the time, and all of the time)* reflecting the frequency of experiencing each feeling in the past 4 weeks. All affirmative responses were counted and calculated towards a total score. Items of well-being were reversely scored, meaning that higher scores represented higher levels of distress and lower levels of well-being. EFQ scores have previously been shown to be highly correlated with scores on the General Health Questionnaire (*r* = 0.8) and to demonstrate high levels of internal consistency. (r = 0.89; [36, 54].

### Support

Support was assessed by one section of the DAWBA complemented with 6 items from phase one of the BCS. A total list of 11 help/support measures were presented to the parents: support from a teacher; support from family or friends; support from books; support from internet; helpline; self-help group; school nurse; special educational needs staff in school; educational psychologist (PPT); specialized mental health worker; other parts of the health care system; and the child protection services. They were used to indicate whether their children and family at any time had received support regarding their children’s feelings, concentration, and/or behavior. All affirmative responses were counted and calculated as a cumulative *Total support* score. A total score was from 0–11, with a range of 0–3. The higher total score, the more support the child had received.

## Statistical analysis

We conducted descriptive analysis of the sample. Independent samples *t-*tests were used to compare parent reported family stressors, parents’ well-being, parenting practices and support received in children with PTSS vs children with no PTSS. Cohens’ *d* were used as a measure of the effect size of the mean differences across PTSS and covariates [11]. The larger the effect size the stronger the relationship between the variables; *d* ≥ 0.2 is considered a small effect, *d* ≥ 0.5 is considered a medium effect size and *d* ≥ 0.8 a large effect size [11]. The relationship between the frequency of PTEs and posttraumatic symptomatology reported by parents on behalf of their children was assessed by computing a Spearman correlation coefficient. The data were analyzed using IBM SPSS Statistics (Version 26) predictive analytic software.

## Results

Out of the 2043 participants (50.7% female; mean age 12.5, SD = 0.8), parents reported that 4.8% of their children (N = 97; 51.5% female; mean age 12.6, SD = 0.8) had been exposed to PTEs and a total of 1.5% (N = 30; 56.7% female; mean age 12.4, SD = 0.9) were reported to have current PTSS and distress/behaviour change, see Table 1. Out of the 97 participants who were exposed to PTEs, 30% were reported to have current PTSS and current distress/behaviour change. No participants were diagnosed with PTSD. The most frequent PTE the parents reported on behalf of their children was *other severe trauma* (55.7%), while the least frequent PTEs reported were *child experienced physical abuse* (1%) and *child experienced other disasters* (1%).

**Table 1:** Descriptive statistics and types of post traumatic experiences (PTE) (N = 97)

Variable	%	N
Gender		
Male	48.5%	47
Female	51.5%	50
Age	12.6 (SD = 0.8)	
Highest maternal education		
Primary	4.40%	4
Secondary	23.40%	21
College/University	72.60%	66
Don´t know	6%	6.2
Highest paternal education		
Primary	6.90%	6
Secondary	31%	27
College/University	62%	54
Don´t know	10.30%	10
PTEs		
Other severe trauma	55.7%	54
Child experienced serious accident?	19.6%	19
Witnessed accident, sudden death	11.3%	11
Witnessed domestic abuse	6.2%	6
Child experienced sexual abuse	6.2%	6
Child experienced fire	5.2%	5
Child experienced attack or threat	4.1%	4
Witnessed attack	3.1%	3
Child experienced rape	2.1%	2
Child experienced physical abuse	1.0%	1
Child experienced other disasters	1.0%	1

The most frequent post traumatic symptom cluster the parents reported on behalf of their children was *arousal reactivity* (90.0%). This was followed by *negative cognitions and mood* (80.0%), *intrusions* (63.3%) and *avoidance* (60.0%), see Table 2.

Within the cluster of *arousal reactivity,* the most frequently reported symptom was *poor concentration due to stressful event* (76.7%) and the most often reported symptom within the cluster of *negative cognitions and mood* was *blocked out memories due to stressful event* (53.4%). Within the cluster of *intrusions, distress due to stressful event if reminded* (53.3%) was the most common symptom reported. Within the cluster of *avoidance,* the most frequently reported symptom was *avoids thinking or talking about trauma* (53.3%). The descriptive characteristics of PTSS can be found in Table 2.

**Table 2:** Descriptive statistics and post traumatic symptoms (N = 30)

Variable	%	*N*
Gender		
Male	43.3	13
Female	56.7	17
Age, *M (SD)*	12.4 (0.8)	
Distress/behaviour change at the time of exposure	53.6	52
Current distress/behaviour change	30.9	30
*Clusters*		
** Intrusions**	63.3	19
Distress due to stressful event if reminded	53.3	16
Flashbacks due to stressful event	33.3	10
Nightmares due to stressful event	26.7	8
**Avoidance**	60.0	18
Avoids thinking or talking about trauma	53.3	16
Avoids associated activities, places, or people	20.0	6
**Negative cognitions and mood**	80.0	24
Blocked out memories due to stressful event	53.4	16
Feels cut off from others due to stressful event	30.0	9
Loss of confidence in future due to stressful event	26.7	8
Reduced affective range due to stressful event	16.7	5
Lost interest in activities due to stressful event	16.6	5
**Arousal reactivity**	90.0	27
Poor concentration due to stressful event	76.7	23
Irritable/angry due to stressful event	63.4	19
Insomnia due to stressful event	53.4	16
Alert to danger due to stressful event	43.3	13
Easily startled due to stressful event	33.3	10

As seen in Table 3 and figure 2 there was a significant difference in parent reported total family stress score for those children with PTSS (*M* = 4.7, *SD* = 3.5) and those without PTSS (*M* = 2.5, *SD* = 2.5), *t* (86) = − 3.5, *p* = 0.001, *d* = 0.8). This corresponds to a large effect size [11]. Of those children who had experienced PTEs, the biggest discrepancy in terms of family stressors reported by parents of children with PTSS vs parents of children without PTSS was *tension with ex-partner* (a discrepancy of 31.1%), followed by *financial stress* (a discrepancy of 24.5%), while the smallest discrepancies were *time pressure* (a discrepancy of 1.5%), followed by *quarrels between children* (a discrepancy of 3.1%). Interestingly, *work stress* was reported more frequently by parents of children without PTSS (34.3%) than by parents of children with PTSS (20%).

There was a significant difference between parent reported *support* between children with PTSS (*M* = 2.6, *SD* = 2.0) and those with no PTSS (*M* = 1.2, *SD* = 1.9), *t* (86) = − 3.3, *p* = 0.001, *d* = 0.8) (Table 3 and figure 3). This corresponds to a large effect size and indicates that families with children with post traumatic symptoms report to have used more support than those without post traumatic symptoms. Of children with post traumatic symptoms, the most frequent parent reported source of support came from *family or friends* (36.7%), followed by support from *special educational needs staff in school* (30%). The least frequently used source of support was use of *help- lines* (3.3%). The biggest discrepancy in utilised support between PTE exposed children with PTSS vs those without PTSS was help from the *child protective service* (a difference of 23.3%) and a *specialized mental health worker* (22.5%), while the smallest discrepancy was the use of *help- line* (a difference of 3.3%). Support from a *teacher* was reported more frequently on behalf of children without post traumatic symptoms (25.4%) vs those with post traumatic symptoms (20%).

**Table 3:** PTS symptoms and covariates

	PTS symptoms present (N = 30)	PTS symptoms not present (N = 67)	95 CI %	*p*	*d*
	*% (N)*	*% (N)*			
Total family stress score, *(M (SD))*	4.7 (3.5)	2.5 (2.5)	− 3.80– -0.76	0.004	0.8
Total support score, *(M (SD))*	2.6 (2.0)	1.2 (1.9)	− 2.35– -0.59	0.001	0.75
Everyday feelings questionnaire *(M (SD))*	14.5 (5.7)	12.3 (4.5)	− 4.40– 0.12	0.063	0.4
Family Life Questionnaire					
Support and encouragement, *(M (SD))*	10.4 (1.6)	10.9 (1.2)	− 0.22– 1.15	0.18	0.3
Supervision, *(M (SD))*	2.6 (0.5)	2.6 (0.5)	− 0.24– 0.23	0.98	0.02
Rules, *(M (SD))*	4.0 (1.0)	3.9 (1.1)	− 0.61– 0.38	0.65	0.1
Discipline, *(M (SD))*	2.9 (1.4)	2.4 (1.3)	− 1.02– 0.20	0.18	0.4
Special treatment, *(M (SD))*	4.2 (1.5)	4.7 (1.6)	− 0.16– 1.26	0.13	0.4

A summary of post traumatic symptomatology and frequency of PTEs reported by parents on behalf of their children can be found in Table 4. First, there was no significant association between number of PTEs and PTSS *r* = 0.07, *n* = 30, *p* = 0.710. Second, there were no significant associations between number of PTEs and severity of the different symptom clusters (all *p*s > 0.05, see Table 4). There was a significant correlation between the different symptom clusters of *avoidance* and *intrusion r* = 0.39, *n* = 30, *p* = 0.035, *arousal reactivity* and *intrusion r* = 0.46, *n* = 30, *p* = 0.01, *arousal reactivity* and *avoidance r* = 0.41, *n* = 30, *p* = 0.023, arousal reactivity and negative cognitions and mood r = 0.37, n = 30, p = 0.046, suggesting a moderately strong association between these clusters.

**Table 4:** The correlation between number of PTEs and mean PTSD clusters

Variable	*Mean (SD)*	*Pearson's r*			
		1	2	3	4
1. Sum PTEs	1.2 (0.68)				
2. Intrusion	0.44 (.41)	0.10			
3. Avoidance	0.55 (.56)	− 0.05	0.39*		
4. Negative cognitions and mood	0.37 (.43)	− 0.03	0.17	0.35	
5. Arousal reactivity	0.68 (.40)	0.12	0.46*	0.41*	0.37*

* p <.05

## Discussion

The present cross- sectional epidemiological study with parents as informants, investigated the frequency of PTE exposure, PTSS and PTSD in a Norwegian non-clinical sample of school-aged children. We further examined PTSD symptom clusters and how family stressors, parent well- being, parent practices and support seeking were related to reported symptomatology. Out of the total sample, parents reported that 4.8% children had experienced PTEs at some point in their lives and 30.9% of these children had current PTSS.

Children with PTSS reported higher levels of family stressors and to be in contact with more sources of support than those without PTSS.

## Main findings

### Exposure to PTEs

Out of the total sample, parents reported that 4.8% children had experienced PTEs at some point in their lives, which is a lower percentage than previous research on parent- reported PTEs [41]. The most frequent PTE reported was other severe trauma, followed by child experienced a serious accident, witnessed accident/sudden death. This is not identical to previous research findings that the most frequent parent-reported PTEs to be extreme economic hardship, parents divorced/separated and living with someone with an alcohol or drug problem [6, 41]. The discrepancy in findings might be explained by different PTE categories included in different studies. Our study defined PTEs by 11 categories, while the ACE study by Bethell et al. [6] defined PTEs by 9 categories and the NSCH report [41] by 8 categories. Our study did in some way include the categories of both, through the two accumulation categories: child experienced other disasters; and other severe trauma. Nevertheless, as opposed to our study Bethell et al. [6] and NSCH [41] specifically categorized “parents divorced or separated” as a PTE, an event that happens to 40.9% of Norwegian couples [47]. This difference in categorization could result in many children not being included in our study and measurement of post traumatic symptoms hence excluded, which in turn could mask possible existing associations between number of PTEs and mean post traumatic symptoms and clusters reported.

### Frequency of PTSD symptoms

The findings revealed current PTSS in 30.9% of children exposed to PTE, which was 1.5% of the total sample. To our knowledge there are few studies examining parent reported PTSS not restricted to the clinical level of PTSD. Our study nevertheless supports findings by a previous self-report study which revealed that approximately one third of trauma exposed adolescents had experienced PTSS symptoms [48].

The most reported post traumatic symptom cluster was arousal reactivity, followed by negative cognitions and mood. The least frequent symptom clusters were intrusions and avoidance. Our findings are identical to the results by Kerig et al. [29], with arousal reactivity being the most frequent symptom cluster, followed by intrusions and avoidance. This is important knowledge, seeing that those exposed to PTEs may react with different intensity in terms of symptom clusters, which again, might affect how they respond to different kinds of treatments [14, 15]. Arousal reactivity often leads to externalizing symptoms such as overactivity, poor impulse control, irritability, aggression and rage, which again affects its surroundings and consequently are easier picked up on by informants [29]. Intrusions on the other hand are distinct internal processes, often filled with confusion [3]. Those experiencing it may struggle with understanding the origin and reason for such internal processes. In turn, this can make it difficult to articulate intrusions and hence leave family members unaware of this problem. Experiencing an intrusion might nevertheless lead to increased arousal due to distress, a symptom which is more easily observable. In support of this, we found a significant relationship between the different symptom clusters of arousal reactivity and intrusion. Understandably, parents may be more aware of and better able to report symptoms of arousal reactivity than symptoms of intrusions.

In our study, the most frequently reported symptom of arousal reactivity was poor concentration due to the stressful event, which in effect could be one explanation for why literature has argued that children/adolescents suffering from PTSD and PTSS are often misdiagnosed with AD/HD [45].

None of the parents reported post traumatic symptoms in their children that were sufficient to meet full diagnostic criteria for PTSD. The absence of any formal PTSD diagnoses in our sample is not consistent with Alisic et al. [1] who found that 5.1% of children and adolescents exposed to PTE develop PTSD by parent report. A possible explanation for this discrepancy could be that our study included parents of children aged 11- 13 years old, while Alisic [1] included children up to 19 years of age. Hence, the participants in Alisic´s [1] study might be 8 years older the participants in our study and in effect have had several more years of potential trauma exposure. Also, the meta-analysis by Alisic et al. [1] included participants with PTE exposure only (3563 participants), while our study started off with a general population sample and then narrowed it down to those with PTE exposure (97 participants). Differences in the number and age of participants needs to be taken into consideration when interpreting our findings. Further, Alisic´s [1] prevalence rates were derived from a meta-analysis and our findings from direct observation. Naturally this difference in study design may impact the frequency and variety of responses obtained. Lastly, we used the DAWBA assessment interview for diagnoses, while the studies included in the meta-analysis by Alisic et al. [1] used six different assessment tools and did not include DAWBA, (e.g., the Clinician Administered PTSD Scale, Child and Adolescent Version [43] and the Diagnostic Interview for Children and Adolescents – Revised [44]). In effect, the difference in findings could be due, at least in part, to the diagnostic instruments used.

Finally, presence of PTSS but absence of PTSD in our sample may not be that consequential, as previous studies have reported that children with subthreshold PTSD did not differ significantly from children scoring above PTSD threshold in measures of functional impairment and distress [27]. A more precise way of diagnosing PTSD in children and adolescents could be based on the intensity of symptoms and their relationship to functional impairment, rather than on the threshold number of symptoms [2, 27].

### The associations between PTEs and PTSD symptoms

Our results revealed that there was no significant relationship between number of PTEs and PTSD symptoms, nor between number of PTEs and PTSD symptom clusters. The latter finding is not directly comparable to other studies. To our knowledge there are no studies examining the relationship between number of PTEs and mean PTSD clusters in children and adolescents [35]. However, our findings on the number of PTEs and mean PTSD symptoms can be compared to studies exploring the relationship between accumulated exposure to different types of PTEs and total number of symptoms, including PTSD [10] and studies measuring the effects on more general health and quality of life measures after PTEs (i.e. [6, 18]. As opposed to our study these comparable studies have revealed a cumulative effect, with exposure to more PTEs in childhood being associated with increasing symptom complexity, PTSD and poorer health and quality of life in adulthood. A possible explanation for the difference in cumulative effects could be that the study by Cloitre et al. [10] is based on a clinical sample of children recruited at a trauma clinic, while our study is a non- clinical study. The difference findings between our studies might reflect a systematic difference in PTSD symptomatology between two distinct samples. The findings on cumulative effect by Bethell et al. [6] and Flaherty et al. [18] was on broader and more general measures, not PTSD symptomatology in particular, which might widen the possibility of an effect.

### PTSD symptoms, family stressors, parent well- being, parent practices and sources of support

There was a highly significant difference in family stressors between families of children with versus without PTSS, which suggests that those with more symptomatic children also reported higher levels of family stress. This is in line with previous findings that social and family problems were significantly related to the severity of PTSS [14, 58]. Importantly our study is a cross-sectional correlational study and points at associations only. Family stressors might leave a child more vulnerable to develop PTSS due to its effects on parenting skills and the capacity of a parent to be physically and emotionally present for the child following exposure to PTEs [14, 32, 57]. Alternatively, the presence of PTS symptoms in a child might increase levels of stress within the family [12, 58], seeing that child PTS symptoms impacts the family system much more widely than just the child [28, 56]. As an example, parents of children who experience trauma are themselves at risk of developing PTS symptoms [25], and there is an increased financial burden in families with children with special healthcare needs [31, 37].

There were no significant differences in terms of parent well-being and parenting practices between parents with children with PTSS versus those without PTSS. These results are not consistent with previous findings showing that parenting practices and parent well-being affects the development of PTSD and PTS symptoms in children following exposure to PTEs (i.e. [50, 58]. The lack of concordance with previous findings could be due to different categories included when defining PTEs and differences in population samples. While our study included participants who had been subjected to one or more of 11 different PTEs., the study by [50] included only participants having been exposed to interparental violence. It makes sense that there would be a more direct association between parental well-being and parental practices and PTSS in [50] given the potential effects of interparental violence on parental well-being and behavior. The meta-analysis by Wise and Delahanty [58] included children from a broad age range (4–18 years), hence the children with PTSS were at different developmental stages, which could impact if/how PTSS was associated with parental well-being and parenting practices. An important aspect when interpreting our findings on parent well- being and parenting practices is that children not exposed to PTEs were not included.

Parents of children with PTSS reported the child to be in contact with more sources of support than those without PTS. This finding is consistent with previous research revealing associations between posttraumatic help and PTSD/PTSS following exposure to PTEs [13, 22, 53]. However, our cross-sectional correlational study does not provide information on whether support was put in place before or after the PTE. It is therefore not possible from our findings to state if the support is an indicator of an initial vulnerability of the individual experiencing PTS symptoms or if it points at the help needed due to such symptoms. Receiving support while suffering from PTSS might be a reason why the individuals did not reach full criteria for a PTSD diagnosis. Our study does not however, provide us with knowledge as to such a potential buffering effect of support in the development of post traumatic symptoms. Still, the findings clearly state that those with post traumatic symptoms have been in contact with more sources of support compared to those without post traumatic symptoms.

Parents of children with PTSS reported that the child were in more contact with child protection services, specialized mental health care services, and they received more help from family or friends compared to those without PTSS. These findings can suggest, on one hand, that those receiving this type of support were more vulnerable to being exposed to PTE and subsequently developing post traumatic symptoms, or, on the other hand, that those who developed PTSS were more likely to seek out and receive this type of support. Support from a teacher was reported more frequently by those without post traumatic symptoms vs those with post traumatic symptoms, which could be due to several factors. For example, families of children with PTSS might seek more professional help than help from a teacher would constitute, or that children with PTEs who did not seek help from their teacher missed out on important form of buffering and developed more PTS symptoms, or that developing PTS symptoms somehow limits the likelihood of receiving such help from a teacher. The former is supported by our finding that the biggest discrepancy between those with post traumatic symptoms vs those without post traumatic symptoms was support from the child protection system and specialized mental health care services, however it is not possible to conclude as to the reason for this difference in support.

### Strengths and limitations of the study

The strength of our study is our measure a range of PTEs in a large community sample of children, our use of a structured diagnostic interview to measure symptoms of PTSD and PTSD symptom clusters, and the gathering of information about important covariates that may be related to PTE exposure and development of PTSS and PTSD.

Several limitations must nevertheless be considered when interpreting the findings from the current study. A primary limitation is that the study is cross-sectional, and that we therefore are unable to determine the causal association between the variables and covariates that we have included in our analyses. Secondly, although the initial sample was relatively large, the sample who reported PTE and PTSS was small. Furthermore, only parental responses were available for analysis. The responses given might be affected by parents not knowing or reporting all experiences of their children. Having been exposed to PTEs are in many cases associated with secrecy and feelings of shame [49], either because the PTE happens within a family context or because the child due to different reasons might be hiding the PTE from the parent. Subsequently, this can prone parents to withhold or unknowingly omit such information and consequently limit valid reports of children´s actual experiences. Due to the structure of DAWBA, in our study there are no available data on children who´s been exposed to PTEs without their parent’s knowledge. Although the use of parent report is often the basis for screening and assessment of children and adolescents, obtaining reports from children themselves could have strengthened the study [46]. The concluding capacity of the co- variate Parenting practice was limited by the low reliability of two of subscales in the FLaQ, Discipline and Special treatment. Finally, we lacked details on the timing of the PTE exposure, and the included category of *other severe trauma* should have been elaborated on to be more informative.

### Implications for further research

Many children and adolescents are exposed to PTEs. After exposure to a PTE, short term distress is common, however most children return to their prior levels of functioning. For those who develop PTSS this can cause great impairment which may last into adulthood. There is a need for future research that can expand our knowledge on the variety and complexity of children and adolescents´ reactions to PTEs and hence strengthen the field´s understanding of the varied trajectories of children and adolescents´ reactions to, and recovery from PTEs. Both when conducting research and in clinical practice, our findings indicate the need for caution when using parents as only informants when screening for PTEs and posttraumatic symptomatology. It is therefore advised to sample responses from both the child as well as the parent- and statistically check for inter-rater reliability. Future studies are also encouraged to explore posttraumatic symptom clusters in children/adolescents both in Norway and internationally, especially with a focus on how the intensity of struggle within each cluster are related to functional impairment and specific behavioural problems. Furthering this field of trauma study will strengthen our knowledge of how-to tailor treatment techniques to specific posttraumatic symptom profiles.

Future research is also advised to examine if support was put in place before or after the PTE occurred when examining the relationship between support and posttraumatic symptomatology. To date this is not specified in the DAWBA interview and should in a study of PTSS be included as an additional question to the participants confirming PTEs. Overall, research is encouraged to repeat our study, though with a larger sample size, a variety of informants and within different cultures.

## Conclusion

Using data from the population-based Bergen Child Study the present study revealed a lower prevalence rate of PTEs and PTSD than previous studies in the field of trauma. It provided findings on parent- reported PTSS and PTSD symptom clusters not restricted to the clinical level of PTSD. Lastly, it highlighted how family-life stressors and support differed between those who had PTSS and those with no PTSS.

Key takeaway messages for clinicians with the aim of minimizing the likelihood of developing PTSS and PTSD when supporting families after a child has been exposed to PTEs is to pay extra attention to children whose environment contains several/ specific stressors and those who need or already receive formal support. When screening children it is advised to keep in mind the limitations of parents as informants and always endeavor to obtain information obtained directly from the child in question.

## Data Availability

The datasets generated during and/or analyzed during the current study are available from the corresponding author on reasonable request.

## References

[1] Alisic E, Zalta AK, van Wesel F, Larsen SE, Hafstad GS, Hassanpour K, Smid GE (2014). Rates of post-traumatic stress disorder in trauma-exposed children and adolescents: meta-analysis. Br J Psychiatry.

[2] American Psychiatric Association (2013). Post traumatic stress disorder.

[3] Amstadter AB, Aggen SH, Knudsen GP, Reichborn-Kjennerud T, Kendler KS (2013). Potentially traumatic event exposure, posttraumatic stress disorder, and Axis I and II comorbidity in a population-based study of Norwegian young adults. Soc Psychiatry Psychiatr Epidemiol.

[4] Benjet C, Bromet E, Karam EG, Kessler RC, McLaughlin KA, Ruscio AM, Koenen KC (2016). The epidemiology of traumatic event exposure worldwide: results from the World Mental Health Survey Consortium. Psychol Med.

[5] Bethell CD, Newacheck P, Hawes E, Halfon N (2014). Adverse childhood experiences: assessing the impact on health and school engagement and the mitigating role of resilience. Health Aff.

[6] Bisson JI, Berliner L, Cloitre M, Forbes D, Jensen TK, Lewis C, Shapiro F (2019). The international society for traumatic stress studies new guidelines for the prevention and treatment of posttraumatic stress disorder: methodology and development process. J Traumatic Stress.

[7] Brøndbo PHM (2013). Måleegenskaper ved den norske versjonen av development and well-being assessment (DAWBA). PsykTestBarn.

[8] Bøe T, Serlachius AS, Sivertsen B, Petrie KJ, Hysing M (2018). Cumulative effects of negative life events and family stress on children's mental health: the Bergen Child Study. Soc Psychiatry Psychiatr Epidemiol.

[9] Carrion VG, Weems CF, Ray R, Reiss AL. Toward an empirical definition of pediatric PTSD: the phenomenology of PTSD symptoms in youth. J Am Acad Child Adolesc Psychiatry. 2002;41(2):166–73. 10.1097/00004583-200202000-00010. PMID: 11837406. 10.1097/00004583-200202000-00010 11837406

[10] Cloitre M, Stolbach BC, Herman JL, van der Kolk B, Pynoos R, Wang J, Petkova E (2009). A developmental approach to complex PTSD: childhood and adult cumulative trauma as predictors of symptom complexity. J Trauma Stress.

[11] Cohen J, Cohen J (1977). CHAPTER 1 - The concepts of power analysis. Statistical Power Analysis for the Behavioral Sciences.

[12] Connor DF, Ford JD, Arnsten AFT, Greene CA (2014). An update on posttraumatic stress disorder in children and adolescents. Clin Pediatr.

[13] Dai W, Kaminga AC, Tan H, Wang J, Lai Z, Wu X, Liu A (2017). Long-term psychological outcomes of flood survivors of hard-hit areas of the 1998 Dongting Lake flood in China: prevalence and risk factors. PLoS ONE.

[14] Daniunaite I, Cloitre M, Karatzias T, Shevlin M, Thoresen S, Zelviene P, Kazlauskas E (2021). PTSD and complex PTSD in adolescence: discriminating factors in a population-based cross-sectional study. Eur J Psychotraumatol.

[15] Donbaek DF, Elklit A, Pedersen MU (2014). Post-traumatic stress disorder symptom clusters predicting substance abuse in adolescents. Ment Health Subst Use.

[16] Felitti VJ, Anda RF, Nordenberg D, Williamson DF, Spitz AM, Edwards V, Marks JS (1998). Relationship of childhood abuse and household dysfunction to many of the leading causes of death in adults. The adverse childhood experiences (ACE) study. Am J Prev Med.

[17] Felitti VJ, Anda RF, Nordenberg D, Williamson DF, Spitz AM, Edwards V, Marks JS (2019). Relationship of childhood abuse and household dysfunction to many of the leading causes of death in adults: the adverse childhood experiences (ACE) study. Am J Prev Med.

[18] Flaherty EG, Thompson R, Litrownik AJ, Zolotor AJ, Dubowitz H, Runyan DK, Everson MD (2009). Adverse childhood exposures and reported child health at age 12. Academic Pediatrics.

[19] Ford JD, Greene CA, Goldstein S, DeVries M (2017). Posttraumatic stress disorder and acute stress disorder in childhood and adolescence. Handbook of DSM-5 disorders in children and adolescents.

[20] Goodman R, Ford T, Richards H, Gatward R, Meltzer H (2000). The development and well-being assessment: description and initial validation of an integrated assessment of child and adolescent psychopathology. J Child Psychol Psychiatry.

[21] Hafstad GS, Sætren SS, Myhre MC, Bergerud-Wichstrøm M, Augusti E-M (2020). Cohort profile: Norwegian youth study on child maltreatment (the UEVO study). BMJ Open.

[22] Han H, Noh JW, Huh HJ, Huh S, Joo JY, Hong JH, Chae JH (2017). Effects of mental health support on the grief of bereaved people caused by sewol ferry accident. J Korean Med Sci.

[23] Heiervang E, Goodman R (2011). Advantages and limitations of web-based surveys: evidence from a child mental health survey. Soc Psychiatry Psychiatr Epidemiol.

[24] Heiervang E, Stormark KM, Lundervold AJ, Heimann M, Goodman R, Posserud M-B, Gillberg C (2007). Psychiatric disorders in Norwegian 8- to 10-year-olds: an epidemiological survey of prevalence, risk factors, and service use. J Am Acad Child Adolesc Psychiatry.

[25] Hiller RM, Halligan SL, Ariyanayagam R, Dalgleish T, Smith P, Yule W, Meiser-Stedman R (2016). Predictors of posttraumatic stress symptom trajectories in parents of children exposed to motor vehicle collisions. J Pediatric Psychol.

[26] Hughes K, Bellis MA, Hardcastle KA, Sethi D, Butchart A, Mikton C, Jones L, Dunne MP (2017). The effect of multiple adverse childhood experiences on health: a systematic review and meta-analysis. The Lancet Public Health.

[27] Kaminer D, Seedat S, Stein DJ (2005). Post-traumatic stress disorder in children. World psychiatry: official journal of the World Psychiatric Association (WPA).

[28] Kazak AE, Kassam-Adams N, Schneider S, Zelikovsky N, Alderfer MA, Rourke M (2006). An integrative model of pediatric medical traumatic stress. J Pediatr Psychol.

[29] Kerig PK, Vanderzee KL, Becker SP, Ward RM (2012). Deconstructing PTSD: traumatic experiences, posttraumatic symptom clusters, and mental health problems among delinquent youth. J Child Adolesc Trauma.

[30] Kessler RC, Aguilar-Gaxiola S, Alonso J, Benjet C, Bromet EJ, Cardoso G, Koenen KC (2017). Trauma and PTSD in the WHO world mental health surveys. Eur J Psychotraumatol.

[31] Kuhlthau K, Hill KS, Yucel R, Perrin JM (2005). Financial burden for families of children with special health care needs. Matern Child Health J.

[32] Lange AMC, Visser MM, Scholte RHJ, Finkenauer C (2022). Parental conflicts and posttraumatic stress of children in high-conflict divorce families. J Child Adolesc Trauma.

[33] Last A, Miles R, Wills L, Brownhill L, Ford T (2012). Reliability and sensitivity to change of the family life questionnaire in a clinical population. Child Adolesc Mental Health.

[34] Lee SH, Kim EJ, Noh JW, Chae JH (2018). Factors associated with post-traumatic stress symptoms in students who survived 20 months after the sewol ferry disaster in Korea. J Korean Med Sci.

[35] Lewis SJ, Arseneault L, Caspi A, Fisher HL, Matthews T, Moffitt TE, Danese A (2019). The epidemiology of trauma and post-traumatic stress disorder in a representative cohort of young people in England and Wales. The Lancet Psychiatry.

[36] Mann J, Henley W, O'Mahen H, Ford T (2013). The reliability and validity of the everyday feelings questionnaire in a clinical population. J Affect Disord.

[37] McClung N, Glidewell J, Farr SL (2018). Financial burdens and mental health needs in families of children with congenital heart disease. Congenit Heart Dis.

[38] McLaughlin KA, Koenen KC, Friedman MJ, Ruscio AM, Karam EG, Shahly V, Kessler RC (2015). Subthreshold posttraumatic stress disorder in the world health organization world mental health surveys. Biological psychiatry.

[39] Meltzer H, Gatward R, Goodman R, Ford T (2003). Mental health of children and adolescents in Great Britain. Int Rev Psychiatry.

[40] Nilsson D, Nordås E, Pribe G, Svedin CG (2017). Child physical abuse – High school students’ mental health and parental relations depending on who perpetrated the abuse. Child Abuse Negl.

[41] NSCH, (2020). Adverse Childhood Experiences. Retrieved from NSCH Data Brief | June 2020

[42] Overstreet C, Berenz EC, Sheerin C, Amstadter AB, Canino G, Silberg J (2016). Potentially traumatic events, posttraumatic stress disorder, and depression among adults in Puerto Rico. Front Psychol.

[43] Pynoos RS, Weathers FW, Steinberg AM, Marx BP, Layne CM, Kaloupek DG, Schnurr PP, Keane TM, Blake DD, Newman E, Nader KO, & Kriegler JA. (2015) Clinician-Administered PTSD Scale for DSM-5 - Child/Adolescent Version.

[44] Reich W, Leacock N, Shanfield C. (1994) Diagnostic Interview for Children and Adolescents – Revised (DICAR). Washington University

[45] Siegfried C. (2016) *Is it ADHD or child traumatic stress? A guide for Clinicians.* Retrieved from Los Angeles, CA & Durham

[46] Skar AS, Jensen TK, Harpviken AN (2021). Who reports what? A Comparison of Child and Caregivers´ Reports of child trauma exposure and associations to post-traumatic stress symptoms and functional impairment in child and adolescent mental health clinics. Res Child Adolesc Psychopathol.

[47] Statistisk sentralbyrå (2020). Marriage and divorce. Retrieved from Oslo, Norway

[48] Stupar D, Stevanovic D, Vostanis P, Atilola O, Moreira P, Dodig-Curkovic K, Knez R (2021). Posttraumatic stress disorder symptoms among trauma-exposed adolescents from low- and middle-income countries. Child Adolescent Psychiatry Mental Health.

[49] Taylor TF (2015). The influence of shame on posttrauma disorders: have we failed to see the obvious?. Eur J Psychotraumatol.

[50] Telman MD, Overbeek MM, de Schipper JC, Lamers-Winkelman F, Finkenauer C, Schuengel C (2016). Family functioning and children’s post-traumatic stress symptoms in a referred sample exposed to interparental violence. J Fam Violence.

[51] Trickey D, Siddaway AP, Meiser-Stedman R, Serpell L, Field AP (2012). A meta-analysis of risk factors for post-traumatic stress disorder in children and adolescents. Clin Psychol Rev.

[52] Twaite JA, Rodriguez-Srednicki O (2004). Childhood sexual and physical abuse and adult vulnerability to PTSD: the mediating effects of attachment and dissociation. J Child Sex Abus.

[53] Udwin O, Boyle S, Yule W, Bolton D, O'Ryan D (2000). Risk factors for long-term psychological effects of a disaster experienced in adolescence: predictors of post traumatic stress disorder. J Child Psychol Psychiatry.

[54] Uher R, Goodman R (2010). The Everyday Feeling Questionnaire: the structure and validation of a measure of general psychological well-being and distress. Soc Psychiatry Psychiatr Epidemiol.

[55] Weisæth L (2002). Vulnerability and protective factors for posttraumatic stress disorder. Psychiatry Clin Neurosci.

[56] Wilcoxon LA, Meiser-Stedman R, Burgess A (2021). Post-traumatic stress disorder in parents following their child’s single-event trauma: a meta-analysis of prevalence rates and risk factor correlates. Clin Child Fam Psychol Rev.

[57] Williamson V, Creswell C, Fearon P, Hiller RM, Walker J, Halligan SL (2017). The role of parenting behaviors in childhood post-traumatic stress disorder: a meta-analytic review. Clin Psychol Rev.

[58] Wise AE, Delahanty DL (2017). Parental factors associated with child post-traumatic stress following injury: a consideration of intervention targets. Front Psychol.

