# Correction: Exposure to potentially traumatic events and PTSD symptomatology in Norwegian 11–13-year-olds: results from the Bergen Child Study

**DOI:** 10.1186/s13034-023-00600-3

**Published:** 2023-04-20

**Authors:** Annika Skandsen, Liv Sand, Martin H. Teicher, Ove Heradstveit, Tormod Bøe

**Affiliations:** 1 Department of Psychosocial Science, Faculty of Psychology, University of Bergen, Bergen, Norway; 2 Stavanger University Hospital, Gerd Ragna Bloch Thorsens Gate 25, Stavanger, Norway; 3 Regional Centre for Child and Youth Mental Health and Child Welfare, NORCE Norwegian Research Centre, Bergen, Norway; 4 Alcohol & Drug Research, Stavanger University Hospital, Stavanger, Norway; 5 Department of Psychiatry, Harvard Medical School, Boston, MA USA; 6 Developmental Biopsychiatry Research Program, McLean Hospital, Belmont, MA USA

**Correction: Child and Adolescent Psychiatry and Mental Health (2023) 17:32** 10.1186/s13034-023-00578-y

Following publication of the original article [1], the authors identified the errors in Figure 3 and Availability of data and materials section. The correct Fig. 3 and updated Availability of data and materials have been presented with this correction.

**Fig. 3:**
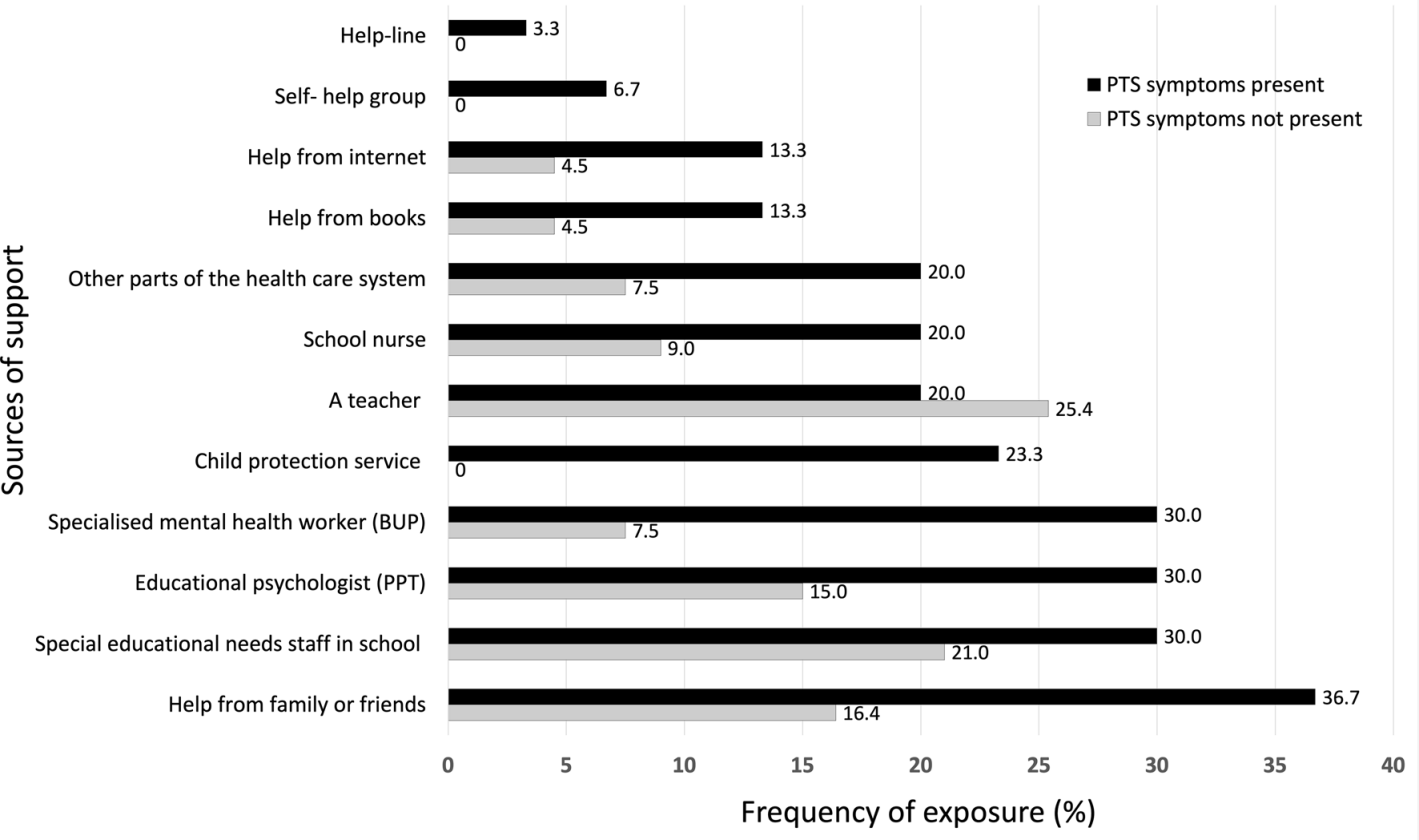
PTS symptoms and use of support

**Availability of data and materials**

Data cannot be shared publicly due to privacy restrictions in accordance with the ethical approval for the Bergen Child Study. Norwegian Health research legislation and the Norwegian Ethics committees require explicit consent from participants in order to transfer health research data outside of Norway. In this specific case, ethics approval is also contingent on storing the research data on secure storage facilities located in our research institution. Data are from the Norwegian Bergen Child Study, owned by NORCE Norwegian Research Centre. The authors did not have special access privileges to data from the survey. Data are available from the Bergen Child Study Institutional Data Access (contact via bib@norceresearch.no) for researchers who meet the criteria for access to confidential data.

The original article has been corrected.
